# The Waxy Gene Has Pleiotropic Effects on Hot Water-Soluble and -Insoluble Amylose Contents in Rice (*Oryza sativa*) Grains

**DOI:** 10.3390/ijms25126561

**Published:** 2024-06-14

**Authors:** Hongkai Wu, Siyuan Wang, Min Wu

**Affiliations:** College of Advanced Agricultural Sciences, Zhejiang A&F University, Hangzhou 311300, China; wangsy@stu.zafu.edu.cn (S.W.); rice@zafu.edu.cn (M.W.)

**Keywords:** rice (*Oryza sativa*) quality, amylose content, amylopectin fine structure, quantitative trait locus (QTL) mapping, pleiotropy

## Abstract

Rice (*Oryza sativa*) is a cereal crop with a starchy endosperm. Starch is composed of amylose and amylopectin. Amylose content (AC) is the principal determinant of rice quality, but varieties with similar ACs can still vary substantially in their quality. In this study, we analyzed the total AC (TAC) and its constituent fractions, the hot water-soluble amylose content (SAC) and hot water-insoluble amylose content (IAC), in two sets of related chromosome segment substitution lines of rice with a common genetic background grown in two years. We searched for quantitative trait loci (QTLs) associated with SAC, IAC, and TAC and identified one common QTL (*qSAC–6*, *qIAC–6*, and *qTAC–6*) on chromosome 6. Map-based cloning revealed that the gene underlying the trait associated with this common QTL is Waxy (*Wx*). An analysis of the colors of soluble and insoluble starch–iodine complexes and their λ_max_ values (wavelengths at the positions of their peak absorbance values) as well as gel permeation chromatography revealed that *Wx* is responsible for the biosynthesis of amylose, comprising a large proportion of the soluble fractions of the SAC. *Wx* is also involved in the biosynthesis of long chains of amylopectin, comprising the hot water-insoluble fractions of the IAC. These findings highlight the pleiotropic effects of *Wx* on the SAC and IAC. This pleiotropy indicates that these traits have a positive genetic correlation. Therefore, further studies of rice quality should use rice varieties with the same *Wx* genotype to eliminate the pleiotropic effects of this gene, allowing the independent relationship between the SAC or IAC and rice quality to be elucidated through a multiple correlation analysis. These findings are applicable to other valuable cereal crops as well.

## 1. Introduction

Improving rice (*Oryza sativa*) quality is a principal aim of rice breeding. Starch accounts for approximately 90% of the dry weight of the grain. The type of starch present in rice grain strongly influences quality [1]. The starch in rice endosperm is composed of two types of glucose polymers: the linear, slightly branched polymer amylose and the highly branched polymer amylopectin (AP) [2]. Amylose content (AC), as a percentage of flour weight rather than starch itself, is widely used to evaluate rice quality. Based on extensive studies, the AC is generally considered to be the principal determinant of rice quality [3]; however, AC alone cannot account for the difference in quality between varieties with similar ACs [4].

Bhattacharya and colleagues observed that the hot water-insoluble amylose content (IAC) differs markedly among different rice varieties, even when the total AC (TAC) is in the same range [4,5,6]. Juliano and colleagues observed that low amylose solubility was associated with a hard gel consistency and low breakdown [7] and very high setback values and faster retrogradation when measured with a Brabender viscograph [8].

The relationship between IAC and rice quality (texture and various other properties) has been thoroughly studied. The stickiness and consistency of cooked rice are strongly correlated with the IAC rather than the TAC [9], as are the viscograph characteristics [10]. The textural properties of cooked rice cannot be explained by the TAC alone, but they are well correlated with the IAC [11]. In addition, the retrogradation degree (%R) was found to be correlated with the IAC [12]. Thus, the IAC rather than the TAC is the key factor determining rice quality [9,10].

In recent decades, many studies have focused on the genetic control of AC. The Waxy (*Wx*) gene, which underlies a major quantitative trait locus (QTL) for AC on chromosome 6 [13,14], encodes granule-bound starch synthase I (GBSSI), which is responsible for amylose biosynthesis in the endosperm [15]. Several genes that give a dull grain phenotype were found to affect the splicing efficiency of the *Wx^b^* allele, which results in low GBSSI activity [16,17,18,19]. An additional 43 QTLs nonallelic to the *Wx* locus have been detected throughout the rice genome (https://archive.gramene.org/db/qtl accessed on 1 December 2023). To date, however, no QTL or gene controlling the IAC has been reported.

In this study, we aimed to identify QTLs for IAC in rice. We leached starch granules from rice flour in a boiling-water bath and separated the sample flour into the hot water-soluble (HWS) and hot water-insoluble (HWI) fractions, allowing us to separate the TAC into the hot water-soluble AC (SAC) and the IAC for further study. We conducted QTL analysis for TAC, SAC, and IAC using two sets of related chromosome segment substitution lines (CSSLs) of rice with a japonica rice cv. Koshihikari (Kosh) as a common genetic background and an indica rice cv. Kasalath or NonaBokra as a donor parent grown in two years. Our findings highlight the pleiotropic effects of *Wx* on SAC and IAC and suggest future avenues of research to further explore and improve grain quality in rice and other crops with starchy endosperm.

## 2. Results

### 2.1. Variation in the TAC, SAC, and IAC in Two Sets of CSSLs

**Figure 1:**
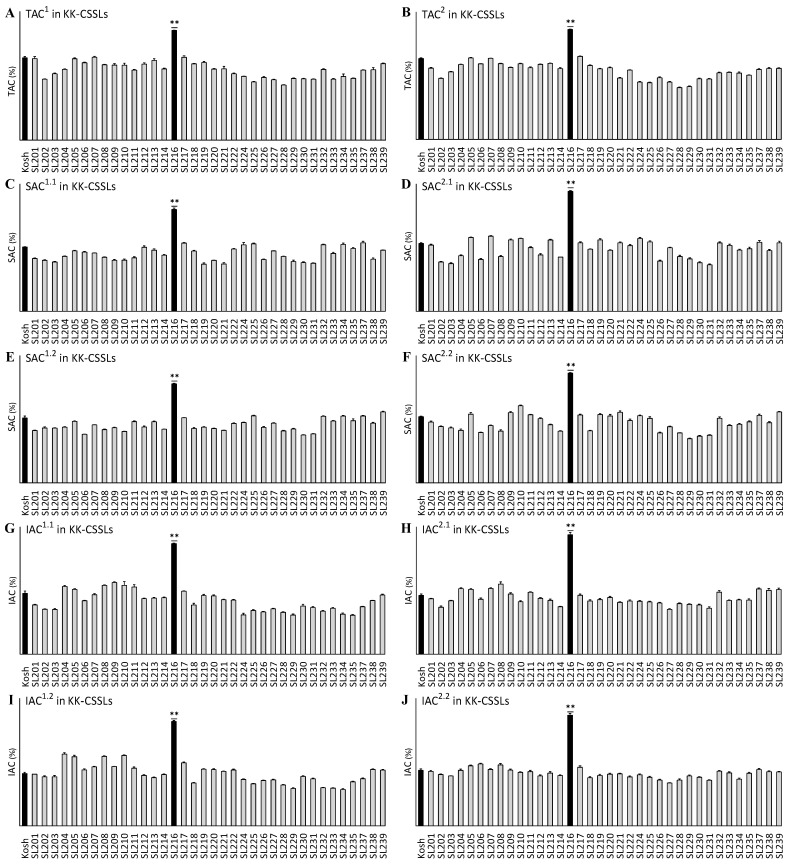
Variation in the total amylose content (TAC), hot water-soluble amylose content (SAC), and hot water-insoluble amylose content (IAC) in the KK-CSSLs including Kosh (Koshihikari, background parent). (**A**,**C**,**E**,**G**,**I**) The KK-CSSLs plants were grown in 2016 and the mean values of TAC, SAC, and IAC exhibited abundant variation, ranging from 11.6% to 16.75%, 8.17% to 12.3%, and 3.43% to 3.68%, respectively. (**B**,**D**,**F**,**H**,**J**) The KK-CSSLs plants were grown in 2017 and the mean values of TAC, SAC, and IAC exhibited abundant variation, ranging from 10.61% to 16.98%, 7.65% to 13.35%, and 4.06% to 5.84%, respectively. The superscript numbers for TAC, SAC, and IAC indicate 2016 or 2017 (“1” or “2” before the decimal point, respectively) and the first or second measurements (“1” or “2” after the decimal point, respectively). For instance, SAC^1.2^ represents the second-measured SAC in 2016. Black bars represent the background parent (Kosh) or line with significantly higher TAC, SAC, or IAC than Kosh using an analysis of variance and the least significance difference test (**, *p* < 0.01). KK-CSSLs, chromosome segment substitution lines with Koshi as the background parent and Kasalath as the donor parent.

We investigated the TAC, SAC, and IAC in two sets of CSSLs grown in two years: 2016 and 2017. Among the Kosh–Kasalath CSSL (KK–CSSL, chromosome segment substitution lines with Koshihikari as the background parent and Kasalath as the donor parent) plants (Figure 1), SL216 had the highest mean TAC, SAC, and IAC values in both 2016 and 2017: TAC (22.19%), SAC (17.39%), and IAC (10.14%) in 2016 and TAC (22.46%), SAC (19.88%), and IAC (10.85%) in 2017. These values were significantly higher than the values of the receptor parent Kosh (*p* < 0.01), although the mean values of TAC, SAC, and IAC in the other lines were similar to those of Kosh, ranging from 11.6% to 16.75%, 8.17% to 12.3%, and from 3.43% to 3.68%, respectively, in 2016, and 10.61% to 16.98%, 7.65% to 13.35%, and 4.06% to 5.84%, respectively, in 2017.

**Figure 2:**
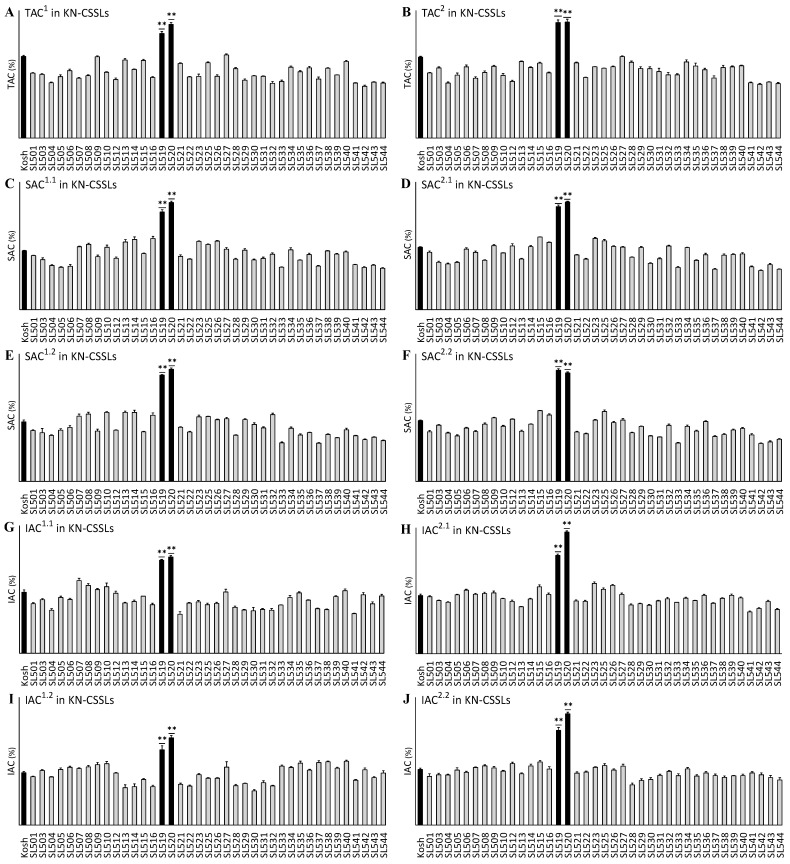
Variation in total amylose content (TAC), hot water-soluble amylose content (SAC), and hot water-insoluble amylose content (IAC) in the KN-CSSLs including Kosh (Koshihikari, background parent). (**A**,**C**,**E**,**G**,**I**) The KN-CSSLs plants were grown in 2016 and the mean values of TAC, SAC, and IAC exhibited abundant variation, ranging from 10.58% to 16.98%, 7.22% to 13.42%, and 3.68% to 6.84%, respectively. (**B**,**D**,**F**,**H**,**J**) The KN-CSSLs plants were grown in 2017 and the mean values of TAC, SAC, and IAC exhibited abundant variation, ranging from 10.99% to 16.65%, 7.17% to 13.7%, and 3.8% to 6.71%, respectively. The superscript numbers for TAC, SAC, and IAC are described in the Figure 1 legend. Black bars represent the background parent (Kosh) or lines with significantly higher TAC, SAC, and IAC than Kosh using an analysis of variance and the least significance difference test (**, *p* < 0.01). KN-CSSLs, chromosome segment substitution lines with Kosh as the background parent and NonaBokra as the donor parent.

Among the Kosh–NonaBokra CSSL (KN–CSSL, chromosome segment substitution lines with Koshihikari as the background parent and NonaBokra as the donor parent) plants (Figure 2), SL519 and SL520 had significantly higher TAC, SAC, and IAC values than Kosh (*p* < 0.01), with mean values of 22.27%, 19.95%, and 8.29%, respectively, in 2016, and 23.63%, 20.29%, and 9.82%, respectively, in 2017. However, the mean values of TAC, SAC, and IAC in the other lines were similar to those of Kosh, ranging from 10.58% to 16.98%, 7.22% to 13.42%, and 3.68% to 6.84%, respectively, in 2016, and from 10.99% to 16.65%, 7.17% to 13.7%, and 3.8% to 6.71%, respectively, in 2017. These results indicate that QTLs for TAC, SAC, and IAC likely exist in the substituted segment of SL216, SL519, and SL520.

### 2.2. Mapping and Identifying QTLs for TAC, SAC, and IAC

In the KK–CSSL set, one common QTL was found to be associated with all three traits (*qTAC–6–1*, *qSAC–6–1*, and *qIAC–6–1* for TAC, SAC, and IAC, respectively). This QTL is located in the interval of the distal end–G200 on chromosome 6. By contrast, no QTL controlling any of these traits was detected in other genomic regions in either year of the study (Table 1). Similarly, in the KN–CSSL set, one common QTL (*qTAC–6–2*, *qSAC–6–2*, and *qIAC–6–2*) was detected in the interval of the distal end–RM5963 on chromosome 6, while no QTL controlling the TAC, SAC, or IAC was detected in other genomic regions in either year (Table 1). The shared genomic regions of these QTLs for each CSSL set suggest that the same genomic region controls the TAC, SAC, and IAC.

**Figure 3:**
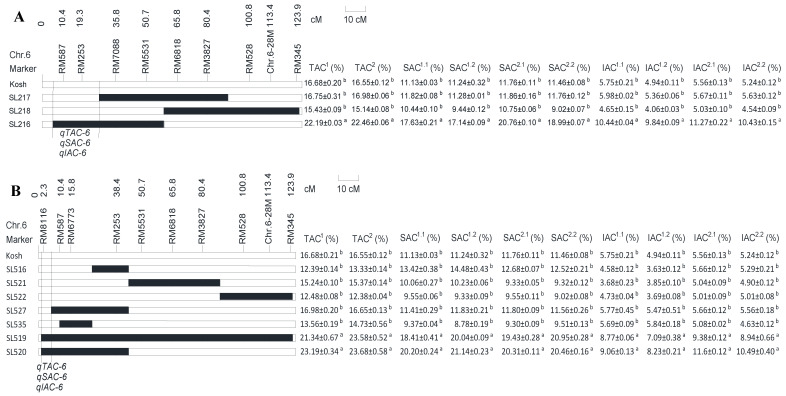
Substitution mapping of the quantitative trait loci (QTLs) *qTAC-6*, *qSAC-6*, and *qIAC-6* on chromosome 6. (**A**) The *qTAC–6*, *qSAC–6*, and *qIAC–6* were mapped to the interval of the distal end–RM7088 on chromosome 6 in the KK–CSSLs. (**B**) The *qTAC–6*, *qSAC–6*, and *qIAC–6* were mapped to the interval of the distal end–RM587 on chromosome 6 in the KN–CSSLs. The solid bars represent chromosome segments from the donor parent; the open bars represent chromosome segments from the background parent (Kosh). Different lowercase letters indicate that the mean values significantly differ (*p* < 0.01), while the same letter indicates no significant difference at *p* > 0.1 using an analysis of variance and the least significance difference test. The superscript numbers for TAC, SAC, and IAC are described in the Figure 1 legend.

To finely map the QTL on chromosome 6, we used polymorphic simple sequence repeat (SSR) markers to construct linkage maps for the KK–CSSLs (Figure 3A) and KN–CSSLs (Figure 3B). Using substitution mapping, *qTAC–6*, *qSAC–6*, and *qIAC–6* were mapped to the interval of the distal end–RM7088 (0–6,649,590 bp) on chromosome 6 in the KK–CSSLs (Figure 3A) and of the interval at the distal end-RM587 (0–2,292,076 bp) on this chromosome in the KN–CSSLs (Figure 3B). These regions overlap with the known *Wx* locus (1,765,622–1,770,653 bp) (https://rapdb.dna.affrc.go.jp/ accessed on 15 December 2023); therefore, we reasoned that the common QTL might be the *Wx* locus.

Three major alleles of the *Wx* gene are present in cultivated rice: *Wx^a^*, *Wx^b^*, and *wx*. *Wx^a^*, which confers high AC, is mainly distributed in *indica* rice. This allele contains a G base at the 5′ first splice donor site of the first intron. *Wx^b^*, which confers low AC, is mainly distributed in *japonica* rice. This allele has a G-to-T mutation at the 5′ first splice donor site of the first intron, which decreases its expression relative to *Wx^a^* [20,21]. *wx* is a loss-of-function allele that produces waxy (opaque) endosperm lacking amylose, which is distinct from the semitransparent endosperm of rice containing *Wx^a^* or *Wx^b^*. The G/T polymorphism can be genotyped using the cleaved amplified polymorphic sequence marker PCR–AccI [22]. We used the PCR–AccI marker to genotype Kosh and the CSSLs (SL216, SL519, and SL520, each harboring the common QTL), which revealed that Kosh contains the +1T nucleotide while SL216, SL519, and SL520 each contain the +1G nucleotide. The G/T polymorphism suggests that the common QTL is identical to the *Wx* locus.

**Figure 4:**
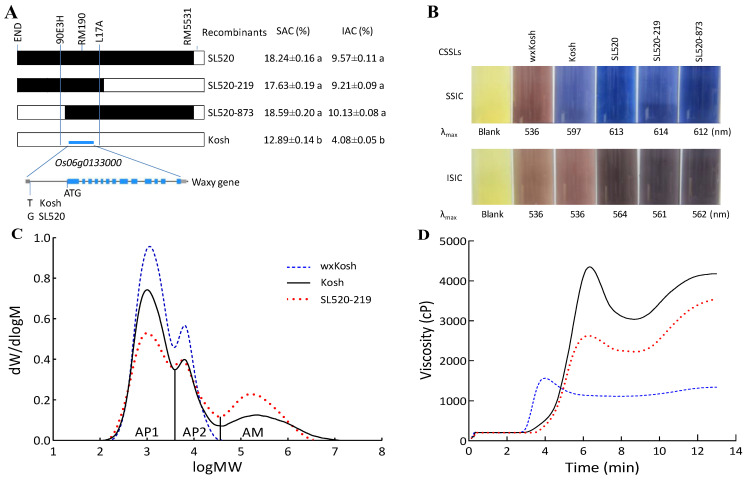
Identification of quantitative trait loci (QTLs) for hot water-soluble amylose content (SAC) and hot water-insoluble amylose content (IAC). (**A**) Fine-mapping of *qSAC-6* and *qIAC-6* using *a* set of smaller chromosome segment substitution lines (CSSLs) with their genotypes and phenotypes. The CSSLs were constituted by the recombinants (SL520-219 and SL520-873) from a cross between SL520 and Kosh. The common QTL (*qSAC-6* and *qIAC-6*) labeled from END and RM5531 was verified as the Waxy gene. The structural diagram of *Wx* was downloaded from https://rapdb.dna.affrc.go.jp/locus/?name=Os06g0133000 accessed on 30 December 2023. The different letters (a and b) mean that their genotypic means significantly differ at *p* < 0.01. (**B**) A composition analysis of the hot water-soluble (HWS) and hot water-insoluble (HWI) fractions of starch. The HWS fractions are composed of amylose and a small quantity of short-chain AP while the HWI fractions are composed of long-chain AP. SSIC, soluble starch–iodine complex; ISIC, insoluble starch–iodine complex. (**C**) Gel permeation chromatograms of isoamylase-debranched starch from wxKosh, Kosh, and SL520–219, which compose a set of near-isogenic lines (NILs) of the *Wx* gene corresponding to the *wx*, *Wx^b^*, and *Wx^a^* alleles, respectively, in the Kosh background. AP1, AP2, and AM correspond to short branch chains of amylopectin, long branch chains of amylopectin, and amylose, respectively. (**D**) Rapid viscosity profiles of flour from the set of NILs exhibited distinct pasting properties at 12% solids content. The legends of lines are the same as (**C**).

To validate this notion, we developed an F_2_ subpopulation by crossing SL520 and Kosh and assessed three markers, 90E3H (1,760,297–1,760,569 bp, upstream of the *Wx* locus), RM190 (1,765,625–1,765,746 bp, inside the *Wx* locus), and L17A (1,770,738–1,770,976 bp, downstream of the *Wx* locus) [23], all of which were polymorphic between SL520 and Kosh. Subsequently, we screened this population for recombinants between the three polymorphic marker loci and obtained two homozygous recombinants (SL520–219 and SL520–873) (Figure 4A). We phenotyped SL520–219 and SL520–873, along with SL520 and Kosh, for SAC and IAC, revealing no significant differences in these traits among SL520, SL520–219, and SL520–873, although all lines had significantly higher SAC and IAC than Kosh (*p* < 0.001). These results indicate that SL520–219 and SL520–873 contain a QTL in their overlapping segments (10.13 kbp genomic region) defined by 90E3H and L17A, in which only the *Wx* locus is present [23]. Therefore, the common QTL (*qSAC–6* and *qIAC–6*) is identical to the *Wx* locus, i.e., *Wx* has pleiotropic effects on the SAC and IAC of rice grains.

### 2.3. Compositions of the HWS and HWI Fractions of Starch

To uncover the mechanism underlying the pleiotropy associated with SAC and IAC, we analyzed the compositions of the HWS and HWI fractions of extracted starch. Amylose–iodine complexes appear blue, while AP–iodine complexes appear brown; thus, we examined the colors of the soluble starch–iodine complex (SSIC) and insoluble starch–iodine complex (ISIC) in wxKosh, Kosh, SL520, SL520–219, and SL520–873 (Figure 4B).

For wxKosh, both the iodine-stained SSIC and ISIC appeared brown, indicating that their HWS and HWI fractions both consisted of AP, with no amylose, which was validated by our GPC results (Figure 4B and Table 2). The iodine-stained SSICs of Kosh, SL520, SL520–219, and SL520–873 were all pure blue, indicating that their HWS fractions contained amylose. The ISIC of Kosh was brown, similar to that of wxKosh, suggesting that its HWI fraction may comprise AP. The ISICs of SL520, SL520–219, and SL520–873 were all dark brown rather than blue, suggesting that their HWI fractions also consisted of AP. Nevertheless, their color (dark brown) was not exactly the same as that of Kosh (brown).

Moreover, we investigated the λ_max_ values of the SSIC and ISIC of wxKosh, Kosh, SL520, SL520–219, and SL520–873 (Figure 4B). For wxKosh, its λ_max_ values for the SSIC and ISIC were both 536 nm. Its HWS fractions accounted for 8.34% of the total OD_536_ value, indicating that the AP of wxKosh is slightly soluble in hot water.

For Kosh, SL520, SL520–219, and SL520–873, their λ_max_ values for SSIC were 597, 614, 613, and 612 nm, respectively, which were slightly lower than that (650 nm) of a purified amylose–iodine complex [24] and much higher than that of AP (536 nm), indicating that, besides amylose, their HWS fractions contained a small amount of AP. Their λ_max_ values for ISIC were 536, 562, 561, and 564 nm, respectively. These results are in agreement with the results reported by Takeda et al. (1987), who showed that the λ_max_ values of purified AP–iodine complexes were ~535 nm from *Wx^b^* varieties and ~565 nm from *Wx^a^* varieties [25]. These results suggest that the HWI fractions of Kosh, SL520, SL520–219, and SL520–873 may consist of AP. The λ_max_ values for the ISIC of SL520, SL520–219, and SL520–873 were higher than that of Kosh. Coupled with the above finding that the ISIC of SL520, SL520–219, and SL520–873 was a darker brown than that of Kosh, these findings highlight the differences in the fine structures of AP between genotypes.

We analyzed the fine structure of starch by determining the relative molecular weight distributions of isoamylase-debranched starch in a set of NILs (wxKosh, Kosh, and SL520–219) using GPC (Figure 4C). The completely debranched starch was fractionated into three components: AP1, consisting of low-molecular-weight molecules, including A and short B chains (A + B1 chains); AP2, consisting of high-molecular-weight molecules, including long B chains; and AM, consisting of a broad range of amyloses [26]. In general, the AM represents the true AC [27].

GPC revealed that wxKosh lacks amylose, while Kosh and SL520–219 have low and high AM (true AC), respectively (Figure 4C; Table 2). These results indicate that *Wx* is responsible for the biosynthesis of amylose. Nevertheless, SL520–219 contained lower levels of long amylose chains than Kosh (Figure 4C), which is in agreement with the findings of Zhang et al. (2012; 2019) [23,28]. This is because amylose contains more branch linkages in the *Wx^a^* vs. *Wx^b^* varieties [29,30].

In terms of the fine structure of AP, the proportion of AP1 decreased, consequently increasing the proportion of AP2 and AM in wxKosh, Kosh, and SL520–219. AP2 and AM govern the texture of cooked rice [31]. The ratio of AP2 to AP1 (AP2/AP1) showed no significant difference between Kosh and wxKosh, whereas this ratio was significantly greater in SL520–219 than in Kosh and wxKosh. This may explain why the color and λ_max_ values of the ISIC of Kosh were the same as those of wxKosh, while the ISIC of SL520, SL520–219, and SL520–873 were darker and their λ_max_ values were higher than those of Kosh and wxKosh (Figure 4C). Thus, the color and λ_max_ value of the ISIC may be regarded as characteristics of the fine structure of AP [25,32]. These results indicate that the *Wx^a^* allele (SL520–219) is more strongly involved in the biosynthesis of long-branch AP chains than *Wx^b^* (Kosh). The APs from the high-AC varieties (carrying the *Wx^a^* allele) contain a high proportion of long B chains [33], which are known to be present in all high-AC cereal starches [34]. In addition, *Wx^a^* is responsible for the biosynthesis of extra-long unit chains of AP. These differences resulted in the structural differences in the AP of the HWI fractions between the *Wx^a^* and *Wx^b^* varieties, and the structural differences contributed to the variation in their IAC (Figure 4B,C).

In conclusion, the HWS fractions are composed of amylose and a small quantity of short-chain AP, while the HWI fractions are composed of long-chain AP. These observations are in agreement with the findings of Bhattacharya et al. (2011) [3] and Butardo et al. (2019) [35]. These findings are also in accordance with the finding by Cuevas et al. (2010) that amylose readily dissolves in hot water while AP is largely insoluble [36]. The *Wx* gene is not only responsible for biosynthesizing amylose, which comprises a large proportion of the HWS fractions that were used to calculate the SAC, but it is also involved in the biosynthesis of long chains of AP, which comprises the HWI fractions that were used to calculate the IAC. As a result, *Wx* has pleiotropic effects on SAC and IAC.

### 2.4. Relationship between SAC or IAC and Quality

The RVA mimics the cooking process of rice. In the RVA, a flour–water suspension is subjected to a heat–hold–cool–hold temperature cycle [37], and the measured values reflect the texture of cooked rice [27]. To elucidate the relationships between SAC or IAC and cooked-rice quality, we measured the flour pasting properties of the lines in an RVA. wxKosh, Kosh, and SL520–219 exhibited distinct pasting properties at 12% solids content (Figure 4D). However, due to the pleiotropic effects of *Wx* on SAC and IAC, these values were positively correlated with each other: the higher the SAC, the higher the IAC, and vice versa (Figure 4A). As a result, the individual relationships between SAC or IAC and the pasting properties of the flour could not be independently determined.

## 3. Discussion

### 3.1. Amylose Content (AC) and Its Relationship with Rice Quality

The AC is the most important physicochemical index of rice quality. Estimating the AC relies on the binding of iodine to amylose to produce a blue amylose–iodine complex, whose absorbance is quantified at 620 nm using a spectrophotometer [38]. This absorbance values also contain the binding of iodine to amylopectin (AP); hence, the AC was designated as the ‘apparent amylose content (AAC)’ [25]. That is, the AC measured based on iodine coloration is a mixture of amylose and AP. In this way, the AC of milled rice grains can be ranked as waxy (0–2% of the flour weight), very low (3–9%), low (10–19%), intermediate (20–25%), or high (>25%) [39].

In this study, by leaching starch granules in a boiling-water bath, we separated the starch samples into the HWS and the HWI fractions, dividing the AC into the SAC and IAC. The HWS fractions are composed of amylose and a small quantity of short-chain AP, and the HWI fractions consist of long-chain AP; thus, the SAC roughly represents the “actual AC”, and the IAC roughly represents the “AP content at 620 nm”. The effect of the AC on rice quality included amylose and AP.

### 3.2. AP Fine Structure and Its Relationship to Quality

Starch granules are composed of amylose and AP [2]. AP is the more abundant and complex of the two components, with both influencing rice quality. In waxy rice, a longer average AP chain length and longer exterior AP chain length result in higher gelatinization temperatures, higher pasting consistencies, greater retrogradation, and a firmer texture of cooked rice [26]. In a study of 11 non-waxy rice cultivars, hard-cooked rice (which was hard after being cooked) tended to have a higher AC and more longer-chain AP than soft-cooked rice [40]. In a study of seven varieties of rice with cooked-rice textures ranging from very soft to very hard, the content of long B chains of AP correlated well with the sensory tenderness of the cooked rice [31]. Thus, variation in the fine structure of AP appears to be at the root of variation in rice texture [31,33,40].

Bhattacharya and colleagues investigated the water solubility of amylose, revealing that the IAC is a key factor in rice quality [9,10]; further research has widely confirmed this finding [12]. In the present study, we determined that the HWI fractions of starch consist of long-chain AP and that structural differences in the AP in the HWI fraction contribute to the observed variation in IAC (Figure 4A–C); therefore, the IAC (AP content at 620 nm) represents variation in the fine structure of AP. That is, IAC can be substituted for AP fine structure to study the impact of the latter on quality.

### 3.3. Pleiotropy of Wx

The starch biosynthesis pathway is a complex network, which requires the involvement of multiple isoforms of some enzymes, including granule-bound starch synthase (GBSS) which is responsible for amylose biosynthesis [15], and soluble starch synthase (SSS), starch-branching enzyme (SBE), and starch-debranching enzyme (DBE) which are together responsible for AP biosynthesis [41,42]. Moreover, ADP-glucose pyrophosphorylase (AGPase) is a rate-limiting step in starch biosynthesis [42]. Each enzyme plays a distinct role, but as parts of this biosynthesis pathway, enzymes (isoforms) involved in AP biosynthesis presumably have a function in amylose biosynthesis [43]. SSSIIIa plays an important part in the elongation of AP chains, and deficiency in the SSSIIIa caused an increase in the amounts of GBSSI which increased amylose content in rice grain [44]. BEIIb is capable of forming the branch linkages in AP biosynthesis, and the knockout of BEIIb led to increased amylose content in rice grain [45,46]. In this study, we showed that GBSSI encoded by *Wx* gene is not only responsible for biosynthesizing amylose but is also involved in the biosynthesis of long chains of AP. These relationships result in pleiotropic effects of starch synthesis-related genes.

As described above, the IAC is a key determinant of cooked-rice quality, which is particularly driven by variation in the fine structure of AP. However, all these findings were based on a small number of research materials, including two types of varieties, high AC (*Wx^a^*) and low AC (*Wx^b^*) varieties, meaning that the effect of *Wx* contributed to the findings. Here, we revealed that *Wx* has pleiotropic effects on the SAC and IAC and that this pleiotropy means that these traits are genetically correlated with each other; thus, the role of IAC in rice quality cannot be determined independently. We, therefore, suggest that further studies of rice quality should use rice varieties with the same *Wx* genotype to eliminate the effect of this gene, enabling the independent relationship between the SAC or IAC and rice quality to be elucidated through a multiple correlation analysis. We believe that this should be a major focus of research because many of the varieties currently cultivated in places such as China have similar TACs but show a huge variation in quality, with no clear pattern of quality characteristics. The current findings could also be applicable to other cereal crops.

## 4. Materials and Methods

### 4.1. Plant Materials

Two sets of related CSSLs obtained from the Rice Genome Resource Center, Japan (http://www.rgrc.dna.affrc.go.jp/stock.html accessed on 15 September 2015) were used in this study. For both CSSLs, Koshihikari (Kosh), a *japonica* rice (*Oryza sativa*) variety with low AC, was used as the common receptor parent, while high-AC *indica* rice variety Kasalath or NonaBokra was used as the donor parent. The Kosh–Kasalath CSSL (KK–CSSL) set comprises 36 lines, although three were lacking in this study (SL215, SL223, and SL236), bringing about a gap of 13.2 cM between the distal end and C390 on chromosome 8. The Kosh–NonaBokra (KN–CSSL) set comprises 39 lines, although five were lacking (SL502, SL511, SL517, SL518, and SL524), bringing about a gap of 19.2 cM between the distal end and RM3308 on chromosome 4. The two sets of CSSLs were grown in Lingshui, Hainan province, China (18° N, 109° E) in two years: during the winter season in November 2016–May 2017 (referred to as 2016 hereafter) and November 2017–May 2018 (referred to as 2017 hereafter).

To fine-map the common QTL that was identified (*qAC–6*, *qAC–6*, and *qAC–6*), an F_2_ subpopulation was developed from a cross between SL520 and Kosh. Its two derived recombinants, SL520–219 and SL520–873 (in the common Kosh background), together with Kosh and waxy Kosh (wxKosh; developed by backcrossing waxy variety Suyuno as the donor parent six times to Kosh as the recurrent parent), possessed the *Wx^a^*, *Wx^a^*, *Wx^b^*, and *wx* alleles, respectively. These lines constituted a set of near-isogenic lines (NILs), which were planted in Linan, Zhejiang province, China (30.16° N, 120.12° E) during the summer season (May–October 2021) for gel permeation chromatography (GPC) and analysis of pasting properties.

All the above plant materials were harvested ~45 days after heading when the seeds had turned yellow. The seeds were air-dried in ambient conditions to ~13% moisture content, stored at room temperature (~25 °C) for three months for after-ripening, and stored in the cold room at 16 °C and <65% relative humidity until use. The seeds were dehulled, milled, ground into flour, and passed through a 0.5 mm sieve before analysis.

### 4.2. Separation of the HWS and HWI Fractions

The HWS and HWI fractions of rice flour were separated as described by Shanthy et al. (1980) [47], with some modifications. Briefly, 0.1000 g milled rice flour was accurately weighed into a 50 mL centrifuge tube. The sample was thoroughly moistened in 1 mL of 95% ethanol, and 20 mL of distilled water was added instead of dilute alkali as a solvent. The samples were vortexed and heated in a boiling-water bath for 30 min with intermittent mixing to fully separate the soluble and insoluble starch. The samples were cooled to room temperature and centrifuged at 5000× *g* for 10 min, and the supernatant was collected in a 100 mL volumetric flask. To fully separate the soluble and insoluble fractions, the separation procedure was repeated one more time with 20 mL of distilled water added to the precipitate. Both supernatants were pooled into the same 100 mL volumetric flask. The supernatant is referred to as the HWS fraction and the precipitate as the HWI fraction hereafter. The flour sample was thus used to calculate the TAC, the HWS fraction was used to calculate the SAC, and the HWI fraction was used to calculate the IAC. Each separation experiment was performed in three biological replications.

### 4.3. Determination of the TAC, SAC, and IAC

The TAC was determined using the iodine colorimetric method, according to EN ISO 6647–1:2020 [48], with minor modifications. Briefly, 0.1000 g of the test sample was mixed with a sodium hydroxide solution, an iodine solution was added for color development, and the absorbance at 620 nm was measured against the blank solution using a spectrophotometer. The SAC and IAC were determined using the method described for the TAC, except that the iodine solution was directly added to the soluble starch fraction for color development. Each sample was determinated in three biological replications.

A calibration line was generated using a concentration series of purified amylose from potato (MilliporeSigma, Burlington, MA, USA) and defatted AP from wxKosh. The calibration line was used to determine the TAC. Another calibration line was generated using a set of standard samples (0.4%, 10.3%, 16.6%, and 26.6%) obtained from the China National Rice Research Institute and used to determine the SAC and IAC. The sample was scanned at a wavelength of 800–400 nm using a UV–visible spectrophotometer (UV2600; Shimadzu, Kyoto, Japan). The λ_max_ represents the wavelength at the position of the peak absorbance value of the iodine–starch complex.

### 4.4. QTL Analysis

QTL analysis was performed using the stepwise regression-based likelihood ratio tests for additive QTL (RSTEP–LRT–ADD) method for CSL (chromosome substitution line) functionality provided by the QTL IciMapping Version 4.1 software package [49], as described by Xu et al. (2023) [50]. A standard threshold logarithm of odds (LOD) score of 3.0 was used to suggest the presence of a putative QTL.

### 4.5. Molecular Weight Distribution of Starch

The molecular weight distribution of starch was determined using an Agilent PL–gel permeation chromatography (GPC) 220 system (Agilent Technologies, Santa Clara, CA, USA), as described by Zhang et al. (2021) [51].

### 4.6. Pasting Properties of Rice Flour

The pasting properties of the samples were determined using a Rapid Viscosity Analyzer (RVA; Model 3D; Newport Scientific, Warriewood, Australia) according to the approved method 61–02 (AACC 2000) described by Wu et al. (2020) [52].

### 4.7. Data Analysis

Analysis of variance (ANOVA) was performed to compare the genotypic mean TAC, SAC, and IAC values using the least significance difference (LSD) test. Data analysis was performed using SPSS Statistics version 17.0 software for Windows (IBM, Armonk, NY, USA).

## 5. Conclusions

Amylose content (AC) is the principal determinant of rice quality, but varieties with similar ACs still vary in their palatability. In this study, by analyzing the ACs and their constituents, hot water-soluble AC (SAC) and hot water-insoluble AC (IAC), a common QTL (*qAC–6*, *qSAC–6*, and *qIAC–6*) on chromosome 6 associated with all three AC components was revealed to be Waxy (*Wx*) gene. The finding highlights the pleiotropic effects of *Wx* on the SAC and IAC. It implies that further studies of rice quality should be performed using rice varieties with the same *Wx* genotype in order to eliminate the pleiotropic effects of this gene, which could enable the elucidation of the independent relationship between the SAC or IAC and rice quality via a multiple correlation analysis.

## Figures and Tables

**Table 1:** Detection of quantitative trait loci (QTLs) associated with total amylose content (TAC), hot water-soluble amylose content (SAC), and hot water-insoluble amylose content (IAC). Here, 2016.1 and 2016.2 are the first and second detection in plants grown in 2016, respectively; here, 2017.1 and 2017.2 are the first and second detection in plants grown in 2017, respectively. LOD, logarithm of odds; ADD, denotes the additive effect of substituting a donor (Kasalath or NonaBokra) allele for the corresponding receptor (Kosh) allele; M(QQ) and M(qq), mean values of the QQ genotype from the donor and qq genotype from the receptor at the QTL; PVE, phenotype variation explained by the QTL.

Populations	Traits	Years	QTLs	Chr.	Intervals	LOD	M(QQ) (%)	M(qq) (%)	ADD (%)	PVE (%)
KK-CSSL	TAC	2016	*qTAC-6-1*	*6*	end-G200	3.04	22.19	14.04	4.07	41.7
		2017	*qTAC-6-1*	*6*	end-G200	3.78	22.46	13.70	4.38	48.8
	SAC	2016.1	*qSAC-6-1*	*6*	end-G200	3.61	17.63	9.95	3.84	47.2
		2016.2	*qSAC-6-1*	*6*	end-G200	3.15	17.14	9.74	3.70	42.7
		2017.1	*qSAC-6-1*	*6*	end-G200	4.69	20.77	10.59	5.09	45.1
		2017.2	*qSAC-6-1*	*6*	end-G200	3.71	18.99	10.07	4.46	37.8
	IAC	2016.1	*qIAC-6-1*	*6*	end-G200	4.77	10.65	5.74	2.45	41.0
		2016.2	*qIAC-6-1*	*6*	end-G200	3.80	9.84	4.60	2.62	49.0
		2017.1	*qIAC-6-1*	*6*	end-G200	10.57	11.98	5.91	3.03	66.0
		2017.2	*qIAC-6-1*	*6*	end-G200	14.35	10.43	4.91	2.76	84.1
KN-CSSL	TAC	2016	*qTAC-6-2*	*6*	end-RM5963	6.37	22.27	13.02	4.62	57.8
		2017	*qTAC-6-2*	*6*	end-RM5963	8.64	23.63	13.99	4.82	70.1
	SAC	2016.1	*qSAC-6-2*	*6*	end-RM5963	4.32	19.30	10.18	4.56	44.3
		2016.2	*qSAC-6-2*	*6*	end-RM5963	4.56	20.59	10.02	5.29	46.1
		2017.2	*qSAC-6-2*	*6*	end-RM5963	7.00	20.71	9.45	5.63	56.3
		2017.1	*qSAC-6-2*	*6*	end-RM5963	5.34	19.87	9.93	4.97	46.8
	IAC	2016.1	*qIAC-6-2*	*6*	end-RM5963	5.33	8.92	4.89	2.01	44.1
		2016.2	*qIAC-6-2*	*6*	end-RM5963	3.57	7.66	4.82	1.42	38.5
		2017.1	*qIAC-6-2*	*6*	end-RM5963	14.28	11.15	5.80	2.67	75.5
		2017.2	*qIAC-6-2*	*6*	end-RM5963	14.70	9.72	4.93	2.39	82.4

**Table 2:** Gel permeation chromatography parameters of the starches of wxKosh, Kosh, and SL520-219. Data are the means ± standard deviations (SD) (n = 2). AP1, low-molecular-weight molecules, including A and short B amylopectin (AP) chains; AP2, high-molecular-weight AP molecules, including long B chains; AP1/AP2, the ratio of AP2 to AP1; AM, a broad range of amyloses. Further details are provided in the Figure 3 and Figure 4C legends. The different letters (a, b and c) mean that their genotypic means significantly differ at *p* < 0.01.

NILs	AP1	AP2	AM	AP2/AP1
wxKosh	72.44 ± 0.48 a	27.56 ± 0.48 a	0	0.3804 ± 0.009 b
Kosh	59.68 ± 0.37 b	22.61 ± 0.43 c	17.71 ± 0.06 b	0.3788 ± 0.010 b
SL520-219	45.52 ± 0.46 c	26.40 ± 0.20 b	28.08 ± 0.96 a	0.5799 ± 0.005 a

## Data Availability

The data presented in this study are available on request from the corresponding author.
